# The Energetics during the World's Most Challenging Mountain Ultra-Marathon—A Case Study at the Tor des Geants®

**DOI:** 10.3389/fphys.2017.01003

**Published:** 2017-12-05

**Authors:** Aldo Savoldelli, Alessandro Fornasiero, Pietro Trabucchi, Eloisa Limonta, Antonio La Torre, Francis Degache, Barbara Pellegrini, Grégoire P. Millet, Gianluca Vernillo, Federico Schena

**Affiliations:** ^1^Department of Neurosciences, Biomedicine and Movement Sciences, University of Verona, Verona, Italy; ^2^CeRiSM, Research Centre ‘Sport, Mountain and Health', University of Verona, Rovereto, Italy; ^3^Department of Biomedical Sciences for Health, Università degli Studi di Milano, Milan, Italy; ^4^Department of Health Research, University of Health Sciences, University of Applied Sciences and Arts Western Switzerland, Lausanne, Switzerland; ^5^ISSUL, Institute of Sport Sciences, University of Lausanne, Lausanne, Switzerland; ^6^Human Performance Laboratory, Faculty of Kinesiology, University of Calgary, Calgary, AB, Canada

**Keywords:** energy expenditure, locomotion, metabolic cost, mountain ultra-marathon, MUM, ultra endurance, ultra trail, uphill

## Abstract

**Purpose:** To provide insights into the energy requirements as well as the physiological adaptations of an experienced 50-year-old ultra-marathon male athlete during the world's most challenging mountain ultra-marathon (MUM).

**Methods:** The international race supporting the study was the Tor des Geants®, characterized by 330 km with +24,000 m D+ to be covered within 150 h. Before the MUM, we assessed the peak oxygen uptake ($\dot{V}O_{2\text{peak}}$) by means of an incremental graded running test. During the MUM we monitored six ascents (once per race day) with a portable gas analyzer, a GPS and a finger pulse oximeter. We then calculated the net metabolic cost per unit of distance (C), the vertical metabolic cost (C_vert_) and the mechanical efficiency of locomotion (Eff_mech_) throughout the six uphills monitored. We further monitored the distance covered, speed, altimetry and D+ from the GPS data as well as the pulse oxygen saturation with the finger pulse oximeter.

**Results:** Subject's $\dot{V}O_{2\text{peak}}$ was 48.1 mL·kg^−1^·min^−1^. Throughout the six uphills investigated the mean exercise intensity was 57.3 ± 6.0% $\dot{V}O_{2\text{peak}}$ and 68.0 ± 8.7% HR_peak_. C, C_vert_ and Eff_mech_ were 11.4 ± 1.9 J·kg^−1^·m^−1^, 57.9 ± 15.2 J·kg^−1^·${\text{m}}_{\text{vert}}^{-1}$, and 17.7 ± 4.8%, respectively. The exercise intensity, as well as C, C_vert_, and Eff_mech_ did not consistently increase during the MUM.

**Conclusions:** For the first time, we described the feasibility of assessing the energy requirements as well as the physiological adaptations of a MUM in ecologically valid environment settings. The present case study shows that, despite the distance performed during the MUM, our participant did not experience a metabolic fatigue state. This is likely due to improvements in locomotor efficiency as the race progressed.

## Introduction

Ultra-marathon events generally refer to any foot race involving distances longer than the classic marathon length of 42.195 km (Millet and Millet, 2012). During these events, the energy demand is likely to be at the extremes of human tolerance (Millet and Millet, 2012) and the knowledge of this aspect is still scarce (Hill and Davies, 2001; Ardigò and Capelli, 2012). This is likely due to the fact that to date there are no methods available to directly measure the energy demand over long periods of time without placing major (or minor) restrictions on the athletes' activity. However, the knowledge of the energy demand is important for ultra-marathons in general and, particularly, for extreme mountain ultra-marathon (MUM), where the ultra-long distance is coupled to run and/or walk on mountain trails with considerable positive and negative elevation changes and in a harsh mountainous environment (Vernillo et al., 2014, 2015). This information is essential to increase our understanding of the control of human locomotion resulting from the ability to appropriately regulate locomotor behavior in response to change in grade (Vernillo et al., 2016a).

Recently, a growing body of literature on the physiological and biomechanical changes associated with MUM has been published (for example, Millet et al., 2011; Morin et al., 2011; Saugy et al., 2013; Vernillo et al., 2014, 2015; Degache et al., 2015). For instance, our group (Vernillo et al., 2014) showed that after the world's most challenging MUM the energy cost of uphill running decreased by ~14%, due to changes in the uphill-running step mechanics that likely lead to a more economical running style. This improvement in the energy cost of uphill running has been confirmed by another study (Vernillo et al., 2016c). However, these studies investigated only pre-to-post changes. This aspect does not allow the understanding of the evolution of the energy demand during a MUM. Further, for this kind of competition most of the athletes tend to walk for longer sections of the race (Millet et al., 2011; Saugy et al., 2013; Vernillo et al., 2014; Degache et al., 2015). Thus, no physiological data set that provides insight on the influence of slope on the energy cost of walking measured during a MUM is available. Moreover, due to the difficulty to measure the energetics during the competitions (Vernillo et al., 2016c), research has been restricted to laboratory or non-mountainous outdoor settings. Thus, there are no data examining the effect of MUM in ecologically valid environments, thereby limiting our understanding on the physiological changes associated with MUM.

Accordingly, we report for the first time the case of an experienced MUM athlete who participated in the world's most challenging MUM (Saugy et al., 2013; Vernillo et al., 2014; Degache et al., 2015), with the aim to provide preliminary insights into the energy requirements as well as the physiological adaptations during the race.

## Materials and methods

### Participant

Our participant was an experienced 50-years-old MUM male athlete (69.0 kg body mass, 1.73 m body height, BMI of 22.3 kg·m^−2^). Before the race, a questionnaire was administered to collect data about his training experience (Vernillo et al., 2016b). Our participant had 15 years of training in running and 8 years of ultra-marathons experience. Pre-race, weekly training consisted of 3-5 sessions comprising 12.1 ± 3.5 h and 70 ± 19 km with a cumulative elevation change between 5,000 and 10,000 m. Our participant had also a broad experience of MUM races, for example having finished three of the four editions of the present MUM at the time of data collection (2013 edition). Our participant raced this MUM with the aim to finish it in the fastest time possible. Written informed consent was obtained from him. Further, the study was approved by the institutional ethics committee of the Department of Neurosciences, Biomedicine and Movement Sciences of the University of Verona and it was performed according to the ethical standards laid out in the 2013 revision of the Helsinki Declaration for experimentation on human subjects.

### The event

**Figure 1:**
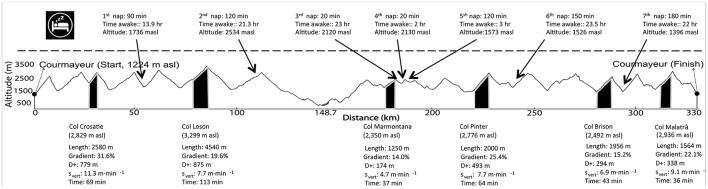
The altimetry and course of the Tor des Geants 2013®. The upper part is about sleeping behavior (time awake refer to the time occurred since last nap). The lower part encloses data about the six uphills monitored.

The international race supporting the study was the Tor des Geants®, characterized by 330 km with a cumulative elevation gain (D+) of +24,000 m that must be completed within 150 h. The altitude along the course ranges between 322 and 3,300 meters above sea level, with 20 mountain passes over 2,000 m (Figure 1).

### Methodology

Peak oxygen uptake ($\dot{V}O_{2\text{peak}}$) was assessed by means of an incremental graded running test performed on a motorized treadmill in a custom-recreated laboratory in Courmayeur (Italy, 1,234 m asl). Breath-by-breath gas exchanges were measured throughout the test by means of a portable metabolimeter (K4b2, Cosmed, Rome, Italy) that was also able to collect the heart rate (HR) data. After apparatus calibration, gas exchanges were measured at rest with the participant standing in an upright position for 5-min. Then, the test started at 6 km·h^−1^ at a constant slope of +10% for 3-min, with speed increments of 0.5 km·h^−1^ every 0.5 min until exhaustion. $\dot{V}O_{2\text{peak}}$ was considered as the highest 30-s $\dot{V}O_{2}$ average during the incremental tests.

During the race we monitored six ascents (Figure 1), once per race day, in which the participant was equipped with the same portable metabolimeter used for the incremental test, a GPS (Forerunner 310XT, Garmin, Olathe, USA) and a finger pulse oximeter (Wristox 3100TM with 8000SM-WO Sensor, Nonin, Plymouth, MN). The setting was complicated because at least 2 researchers were involved on each uphill in order to carry the material required for the data collection and, also, to provide the adequate assistance to the participant. Distance covered, speed (s), altimetry and D+ were obtained from the GPS data. Vertical speed (s_vert_) was calculated as the ratio between D+ and the time to complete each ascent. Percentage of slope gradient was determined from the ratio between D+ and the projection of distance covered on each single ascent. The net metabolic cost per unit of distance (C) was calculated from the ratio between the difference in $\dot{V}O_{2}$ at steady state minus $\dot{V}O_{2}$O_2_ at rest and the speed maintained during each ascent. This value was expressed in J·kg^−1^·m^−1^ by converting the net $\dot{V}O_{2}$ to the corresponding metabolic energy output using an energy equivalent of O_2_ ranging from 21.13 to 19.62 kJ·L^−1^, depending on the RER. The vertical metabolic cost (C_vert_, J·kg^−1^·${\text{m}}_{\text{vert}}^{-1}$) and the mechanical efficiency of locomotion (Eff_mech_) were further calculated (Minetti et al., 2002).

### Statistical analysis

Data are presented as mean ± standard deviation. Given the nature of the study, only a qualitative analysis was performed. To describe the participant's exercise intensity, $\dot{V}O_{2}$ and HR recordings were expressed relative to the participant's peak values (%$\dot{V}O_{2\text{peak}}$ and %HR_peak_, respectively) observed during the incremental test. Due to the time it takes to reach a physiological steady-state condition and for the portable metabolimeter to reflect actual C, the first 5-min of each ascent were discarded in the analysis and the remaining sets were averaged. Pulse oxygen saturation (SpO_2_) was presented as absolute value (i.e., the first and last 5-min blocks).

## Results

There were 705 starters and 383 finishers (54.3%) in the 2013 race. The current record is 67 h 52 min 15 s (set in 2017 edition) and our participant completed the race in 125 h 12 min 19 s (139th place), sleeping for 11 h 40 min (9.3% of the total time). On average, the participant run/walked for 15 h and 40 min without sleeping (range, 2 to 23.5 h). Table 1 summarizes the changes in measurements retrieved during the six uphills. Length, gradient, altitude gain, s_vert_ and duration of each ascent, as well as sleeping behavior, are represented in Figure 1.

**Table 1:** Mean oxygen uptake ($\dot{V}O_{2\text{mean}}$) and heart rate (HRmean) with respect to the peak values (%VO_2peak_ and % HRpeak); net metabolic cost per unit of distance (C); vertical metabolic cost (C_vert_); mechanical efficiency of locomotion (Effmech); Pulse oxygen saturation (SpO_2_) at both the start and the end of each uphill.

**Uphill**	**Altitude at the top (masl)**	**Time from the start (h)**	**VO_2mean_ (%VO_2peak_)**	**HR_mean_ (%HR_pesk_)**	**C (J·kg^−1^·${\text{m}}_{\text{vert}}^{-1}$)**	**C_vert_ (J·kg^−1^·${\text{m}}_{\text{vert}}^{-1}$)**	**Eff_mech_ (%)**	**SpO_2_ start (%)**	**SpO_2_ end (%)**
1	2,825	7	55.5	84.0	11.3	37.4	26.2	94	83
2	3,279	25	66.7	69.2	13.4	69.3	14.1	91	77
3	2,132	57	50.0	62.7	11.1	79.6	12.3	93	91
4	2,266	74	56.9	61.5	13.7	55.5	17.7	92	91
5	1,666	103	52.5	60.9	8.9	59.0	16.6	96	94
6	2,459	120	60.6	69.8	9.8	46.4	21.6	92	88
			57.3 ± 6.0	68.0 ± 8.7	11.4 ± 1.9	57.9 ± 15.2	18.1 ± 5.1	93 ± 1.8	87.3 ± 6.3

*Mean ± SD of the six uphill sections is also reported*.

**Figure 2:**
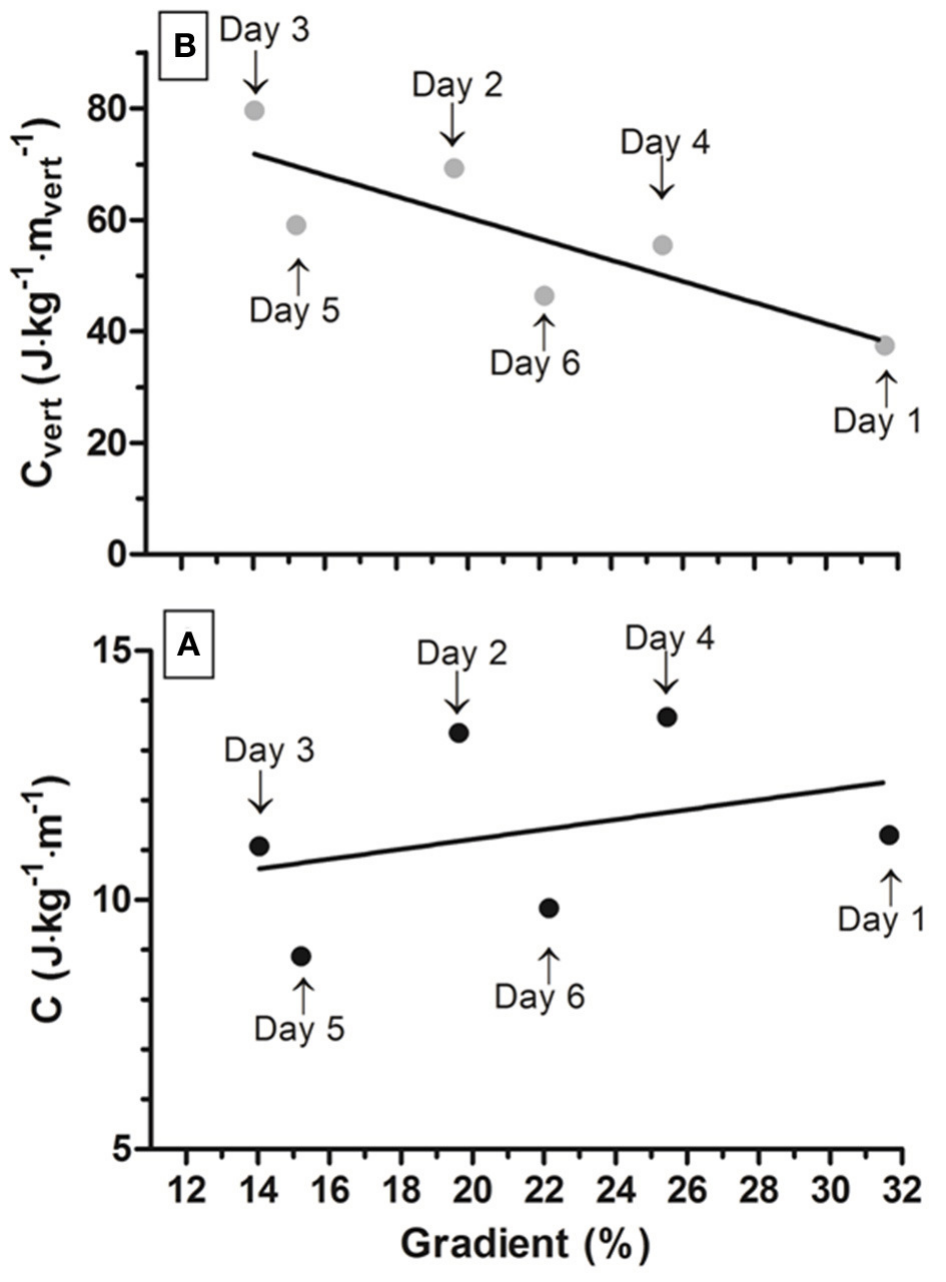
Net metabolic cost (C) and vertical metabolic cost (C_vert_) as a function of the gradient (**A,B**, respectively).

C and C_vert_ as a function of the gradient are presented in Figure 2. Participant's $\dot{V}O_{2\text{peak}}$ and HR_peak_ were 48.1 mL·kg^−1^·min^−1^ and 169 beats·min^−1^ respectively. Throughout the uphills, the mean exercise intensity was 57.3 ± 6.0% of $\dot{V}O_{2\text{peak}}$ and 68.0 ± 8.7% of HR_peak_. Further, mean C and C_vert_ were 11.4 ± 1.9 J·kg^−1^·m^−1^ and 57.9 ± 15.2 J·kg^−1^·${\text{m}}_{\text{vert}}^{-1}$, respectively (Table 1).

## Discussion

To the best of our knowledge, this is the first time that the energy cost of an official finisher of an extreme MUM has been described throughout the race. C and C_vert_ values are in line with the literature, being directly proportional to the slope in the range of gradients here investigated (Minetti et al., 2002; Giovanelli et al., 2015) (Figures 2A,B, respectively). Intuitively, one would assume that the greater distance performed during the MUM, coupled with a fatigue state, should be associated with a drift upwards of the exercise intensity and a rise in the energy cost. However, an intriguing finding of the present study was that the mean exercise intensity, expressed as %$\dot{V}O_{2\text{peak}}$ and %HR_peak_, was similar during the six uphill sections (Table 1). Further, C, C_vert_, as well as Eff_mech_, did not increase throughout the MUM, with the lowest values occurred in the first but also in the last two uphills (Table 1).

Though it can be questionable to generalize the results obtained by investigating a single participant, we can speculate that the improvements found at day 5 and 6 (Figure 2) likely originated from a generic improvement in the mechanical efficiency of locomotion, as already observed in a group of women after a trekking expedition (Tam et al., 2016). Further, this generic improvement in the mechanical efficiency of locomotion could also be responsible for the decreased (improvement) in the uphill energy cost that our group already observed at the end of the same MUM (Vernillo et al., 2014, 2016c). Throughout the MUM, the participant walked during the uphill sections, while adopted a “smoother” running technique during both the flat and downhill sections (authors' personal communication). This strategy likely had the aim to attenuate the overall load faced by his locomotor system at each step (Morin et al., 2011; Millet et al., 2012; Degache et al., 2015). Since uphill locomotion has a movement specific pattern (Vernillo et al., 2016a), this prolonged and repetitive technique likely reflected positive adaptations in the participant's energy requirements and physiological variables during the ~75 h of uphill activity.

## Conclusion

We presented the case of a 50-years-old ultra-marathon athlete with the aim to provide insights into the energy requirements as well as the physiological adaptations during an extreme MUM race. Despite this is a case study and, thus, the generalizability of our findings to all the MUM participants should be done with caution, the present report could be considered a step forward in the analysis of this kind of topic considering the difficulty to apply these measures on the field (Vernillo et al., 2016c). The results indicate that, despite the distance performed during the MUM (i.e., 330 km with +24,000 m D+) the participant did not experience a metabolic fatigue state perhaps due to the prolonged and repetitive locomotion gesture, thereby reflecting a generic improvement in the mechanical efficiency of locomotion (Tam et al., 2016; Vernillo et al., 2016c). These data likely suggest that incorporating long-lasting uphill locomotion training, and hence predisposition to sustaining such loading, is mandatory in the training programs of mountain ultra-marathon athletes to optimize the training process governing this performance.

## Author contibutions

Conceived and designed the experiments: AS, PT, FD, GM, GV and FS. Performed experiments: AS, PT, EL, FD. Analyzed data: AS, AF, GM and GV. Interpreted results of research: AS, AF, PT, EL, AL, GM, GV and FS. Drafted manuscript and prepared tables/figures: AS, AF, EL, BP, GV and FS. Edited, critically revised paper and approved final version of manuscript: AS, AF, PT, EL, AL, FD, BP, GM, GV and FS. All authors have agreed to be accountable for all aspects of the work related to its accuracy and integrity.

### Conflict of interest statement

The authors declare that the research was conducted in the absence of any commercial or financial relationships that could be construed as a potential conflict of interest.
